# Synthesis of New Acadesine (AICA-riboside) Analogues Having Acyclic d-ribityl or 4-Hydroxybutyl Chains in Place of the Ribose

**DOI:** 10.3390/molecules18089420

**Published:** 2013-08-06

**Authors:** Stefano D’Errico, Giorgia Oliviero, Nicola Borbone, Jussara Amato, Vincenzo Piccialli, Michela Varra, Luciano Mayol, Gennaro Piccialli

**Affiliations:** 1 Dipartimento di Farmacia, Università degli Studi di Napoli Federico II, Via D. Montesano 49, Napoli 80131, Italy; E-Mails: stefano.derrico@unina.it (S.D.); nicola.borbone@unina.it (N.B.); jussara.amato@unina.it (J.A.); varra@unina.it (M.P.); mayoll@unina.it (L.M.); picciall@unina.it (G.P.); 2 Dipartimento di Scienze Chimiche, Università degli Studi di Napoli Federico II, Via Cintia 21, Napoli 80126, Italy; E-Mail: vinpicci@unina.it

**Keywords:** AICAR, ZMP, acadesine, AMPK, AMPK activation, imidazole nucleosides, nucleoside analogues, modified nucleosides, acyclic nucleosides, acyclic nucleotides

## Abstract

The antiviral activity of certain acyclic nucleosides drew our attention to the fact that the replacement of the furanose ring by an alkyl group bearing hydroxyl(s) could be a useful structural modification to modulate the biological properties of those nucleosides. Herein, we report on the synthesis of some novel acadesine analogues, where the ribose moiety is mimicked by a d-ribityl or by a hydroxybutyl chain.

## 1. Introduction

A complete understanding of the interactions of the complex metabolic network and of its numerous and in some cases unclear regulator mechanisms still today constitutes a challenge for many researchers in the biological and biomedical fields. This is especially significant if it refers to cells that are affected by diseases and survive with certain modified metabolic pathways. The nucleosides, and their structurally related biomolecules have a very important role in the metabolism acting as synthetic precursors and regulatory agents and being involved in signal transduction. They can be agonists or antagonists of central enzymes in normal or altered metabolic pathways and can be useful tools to demonstrate or affect metabolic rewiring. In most cases they have become important drugs [1,2,3,4,5,6]. In summary they are molecules that stimulate intense research aimed at the development of new structural analogues possessing potential regulatory or pharmacological activities [7,8,9,10,11].

**Figure 1:**
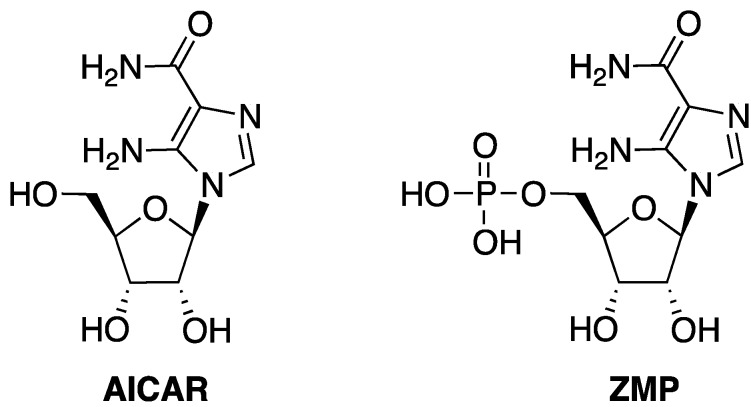
Structures of AICAR and ZMP.

In this context, 5-aminoimidazole-4-carboxamide riboside (acadesine or AICAR, Figure 1) has central role, acting as both a purine biosynthetic precursor and as a modulator of a very high number of biological properties. AICAR, after its 5'-phosphorylation to ZMP, is involved in important metabolic pathways through the activation of the AMP-activated protein kinase (AMPK). In the cells AICAR is phosphorylated to ZMP (Figure 1) that is a mimic of adenosine 5'-monophosphate (AMP) [12,13]. The direct binding of ZMP to an allosteric site of AMPK causes its phosphorylation and activation by a cellular kinase, resulting in a series of important metabolic events, including the inhibition of the basal and insulin-stimulated glucose uptake [14,15], the inhibition of lipid synthesis and the activation of certain ATP-generating processes such as glycolysis and fatty acid oxidation [16]. In the treatment of ischemia, the cardio-protective effect of AICAR has been attributed to the stimulation of the release of extracellular adenosine levels as well as to the activation of AMPK [13,17].

The AMPK pathway is also implicated in the regulation of cell proliferation and activation by AICAR could result in pro-apoptotic effects [18,19]. In particular, AICAR has been revealed to also be an antagonist of the protein Hsp90, a chaperone that regulates the correct interaction between proteins [20]. In such tumors Hsp90 is over-expressed, promoting aberrant cell survival and reproduction even in hostile environments [21].

Recently, it has been established that mutations affecting the Ca^2+^ releasing channel RYR1 are associated with a broad spectrum of human disorders, including malignant hyperthermia, central core disease and core-rod myopathy [22]. By using a mouse model of malignant hyperthermia having a mutation in the RYR1 gene, Lanner *et al*. have recently demonstrated that AICAR can inhibit Ca^2+^ leakage through RYR1 by a mechanism independent from AMPK activation, thus preventing heat-induced sudden death in the mutated mouse [22].

Nevertheless, AICAR is far from being a good drug lead because it has a short half-life in cells and is not strictly specific for the AMPK enzyme; furthermore, it suffers from a number of side effects: it increases uric acid production and favors lactic acidosis [15]. In light of the fact that AICAR can enter into AMPK–dependent or -independent processes [23,24,25], the design and synthesis of novel AICAR derivatives/analogues could be useful to better understand how the related metabolic pathways work and how to obtain new drug candidates.

The discovery of the antiviral activity of acyclovir and the acyclic nucleoside phosphonates [6,26,27] has emphasized that the replacement of the furanose ring by an alkyl group bearing hydroxyl(s) could be an interesting structural modification to induce new properties in the biological activity of the nucleosides. In most cases, the activity of these sugar-modified nucleosides has been attributed to the conformational freedom adopted by the alkyl chain that could, in principle, promote the recognition by the nucleoside related enzymes and prevent the development of viral resistance [27]. Furthermore, the conformational flexibility of acyclic nucleosides and nucleotides could also influence their base-pairing properties. In fact, Van Aerschot *et al*., with the aim of discovering universal nucleoside analogues, have inserted novel acyclic nucleoside derivatives in oligonucleotide strains, evaluating their hybridizing properties [28].

In recent years, we have focused our attention on the preparation of new base- and sugar-modified nucleosides and nucleotides both by classic solution chemistry and, more recently, by a solid-phase approach [29,30,31,32], enlarging the collection of new potential antimetabolites. Herein, we report on the synthesis of a small set of 5-aminoimidazole-4-carboxamides (AICAs) carrying d-ribityl or 4-hydroxybutyl chains at the N1-imidazole position, as well as 5-hydroxypentyl chains at the 4N carboxamide position.

## 2. Results and Discussion

The preparation of imidazole nucleoside analogues having non-glycosidic linkages has usually been accomplished by the condensation of 2-amino-2-cyanoacetate with a suitable chiral or achiral aminoalcohol in the presence of triethylorthoformate, obtaining compounds that show potent adenosine deaminase activities [33]. Other authors have reported on the synthesis of 1-(4-*O*-methyl-2-deoxy-d-ribityl)-5-amino-4-carboxamide imidazole and its derivatives coupling the imidazole portion with a fully protected and activated polyhydroxyalkyl chain by S*_N_*2 displacement [28].

**Scheme 1:**
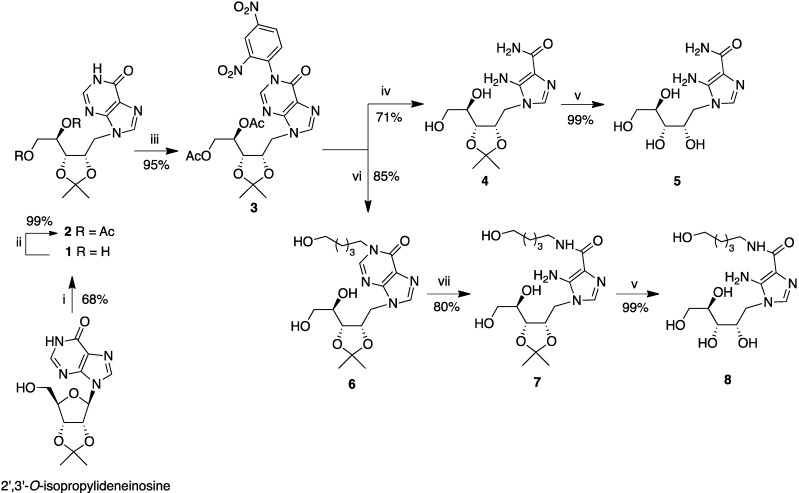
Synthesis of compounds **5** and **8**.

In accordance with Hirota’s procedure [34], in the first part of our work we attempted the preparation of derivative **5** (Scheme 1), in which a ribityl chain replaces the ribose moiety, by the direct reductive cleavage of the C1'-O4' bond of AICAR and of 2',3'-*O*-isopropylidene AICAR. However, this approach failed, resulting only in complex reaction mixtures in both cases. Therefore, we designed a new synthetic route, on the assumption that the construction of this kind of open-ribose AICAR could be performed starting from a suitable purine nucleoside, from which the 5-amino-4-carboxamide moiety could be obtained through the degradation of the purine ring. In the last years we have reported some synthetic strategies to obtain AICAR, ZMP and their 4N-alkyl derivatives, through the purine ring degradation of suitable inosine or 5'-phosphate inosine precursors, carrying a strong electron-withdrawing group (2,4-dinitrophenyl, DNP) at the N1 of the purine ring [29,30,31,32,35].

Considering the instability of the DNP group under the reductive conditions necessary to open the ribose, its introduction at the N1 base position was performed after the reductive cleavage of the C1'-O4' bond of the 2',3'-O-isopropylideneinosine [34] by reaction with DIBAL-H in dry THF that furnished the ribitylinosine **1** in 68% yield. The hydroxyls of **1** were then protected by acetylation, yielding compound **2** (Scheme 1).

*Reagents and conditions*: (i) DIBAL-H, THF, 24 h, r.t.; (ii) Ac_2_O, Py, 16 h, r.t.; (iii) K_2_CO_3_, 2,4-dinitro-chlorobenzene (DNClB), DMF, 3 h, 80 °C; (iv) EDA, DMF, 50 °C, 16 h; (v) 10% TFA, 0 °C then r.t.; (vi) 5-amino-pentan-1-ol, DMF, 50 °C, 16 h; (vii) 5 M NaOH, EtOH, reflux, 5 h.

Compound **2** was then reacted with 2,4-dinitrochlorobenzene (DNClB) in the presence of K_2_CO_3_ to give the intermediate **3**. This reaction served to activate the C2 position of the purine, rendering it susceptible to nucleophilic attack by the amines, which induces the cleavage of the N3-C2 purine bond. In particular, we have previously demonstrated that when a 1,*ω*-diaminoalkane is employed, the fate of the open intermediate depends on the length of the alkyl chain separating the two amino groups. If the diamine is composed of two or three methylene groups, AICAR is formed in a high yield [35]. Therefore, compound **3** was treated with a solution of ethylendiamine (EDA) in DMF and compound **4** was obtained (71% yield) by purine-ring opening/degradation and the concomitant deacetylation of the 4'–5' hydroxyl groups of the ribityl moiety. Finally, the isopropylidene group removal on **4** was performed by 10% trifluoroacetic acid (TFA) treatment, affording the N1-ribityl AICA **5** quantitatively. 

In order to obtain further AICAR derivatives we probed the reactivity of **3** with the 5-amino-pentan-1-ol. As expected this reaction, following a mechanism that starts with the scission of the N3-C2 bond and then proceeds with the reclosure of the purine ring, furnished the N1-hydroxyalkyl-inosine **6** (85% yield). During this reaction a concomitant deacetylation of the hydroxyl groups at 4' and 5' ribityl moiety was observed.

It is well known that N1-alkylated inosines are susceptible to purine ring opening/degradation when treated with alkali [36], affording the corresponding 4N-alkyl AICARs [31,32,35]. In accordance with these data, **6** was refluxed with 5M NaOH solution in ethanol, giving a good yield (80%) of **7**. The reaction was followed by UV spectrophotometry, because TLC monitoring was difficult to perform. After 5 h the disappearance of the purine band at *λ*_max_ 249 nm (pH = 7) and the concomitant appearance of the imidazole band at *λ*_max_ 268 nm (pH = 7) confirmed the end of the reaction. The isopropylidene group from **7** was removed as for **4**, providing **8** in an almost quantitative yield.

The success of reactions **3**→**6**→**7** furnishes the possibility of modulating the length of the hydroxyalkylic chains bound at the N1 (hypoxanthine) or 4N (imidazole-carboxamide) positions, opening the way to the preparation of novel 1-hydroxyalkyl-9-(d-ribityl)-hypoxanthines and 5-amino-1-(d-ribityl)-*N*-(hydroxyalkyl)-imidazole-4-carboxamides, respectively.

**Scheme 2:**
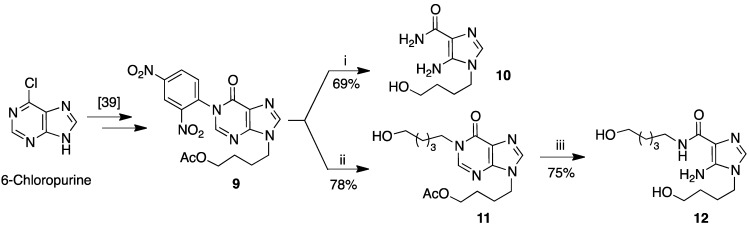
Synthesis of compounds **10** and **12**.

In the second part of our synthetic work we planned to tune a synthetic procedure to obtain AICA derivatives bearing hydroxyalkyl chains at the AICA N1 and at the 4N positions [37,38]. To achieve this goal, we identified 6-chloropurine as a useful precursor on which to introduce the suitable 4-hydroxybutyl chain, followed by its transformation into the AICA derivative **10**. Specifically, in a previous paper we demonstrated that 6-chloropurine could be readily transformed into **9** with a good yield by the following sequence of reaction: base-mediated alkylation on N9, acidic hydrolysis of chlorine, acetylation of the primary hydroxyl and reaction of the resulting hypoxanthine derivative with DNClB (Scheme 2) [39]. As for **3**, the treatment of **9** with EDA in DMF produced the N1-hydroxybutyl AICA **10** in 69% yield.

*Reagents and conditions*: (i) EDA, DMF, 50 °C, 16 h; (ii) 5-amino-pentan-1-ol, DMF, 50 °C, 16 h; (iii) 5 M NaOH, EtOH, reflux, 5 h.

Alternatively, the treatment of **9** with 5-amino-pentan-1-ol produced **11** in a 78% yield, which, unexpectedly, still retained the acetyl group, as detected by spectroscopic analyses. This fact was not detrimental for the completion of the synthesis. In fact, as for **6**, by refluxing **11** with 5 M NaOH solution in ethanol, **12** was obtained (in 75% yield), after the purine ring degradation and hydrolysis of the acetyl group.

The synthesis of compound **12** opened the way to the preparation of novel AICA analogues, combining sugar alterations with base modifications on the natural skeleton of the AICA riboside.

## 3. Experimental

### General Methods

All the reagents were obtained from commercial sources (Sigma-Aldrich, Milano, Italy) and were used without further purification. ^1^H (400 MHz) and ^13^C (100 MHz) spectra were acquired on a Varian Mercury Plus 400 MHz in CD_3_OD or CDCl_3_. Chemical shifts are reported in parts per million (δ) relative to the residual solvent signals: CD_2_HOD 3.31 and CHCl_3_ 7.27 for ^1^H-NMR; CD_3_OD 49.0 and CDCl_3_ 77.0 for ^13^C-NMR. ^1^H-NMR chemical shifts were assigned by 2D NMR experiments. The abbreviations s, bs, d, bd, dd and m stand for singlet, broad singlet, doublet, broad doublet, doublet of doublets and multiplet, respectively. HPLC analyses and purifications were carried out on a Jasco UP-2075 Plus pump equipped with a Jasco UV-2075 Plus UV detector using a 4.8 × 150 mm C-18 reverse-phase column (particle size 5 µm) eluted with a linear gradient of CH_3_CN in H_2_O (from 0 to 100% in 60 min, flow 1.3 mL min^−1^). UV spectra were recorded on a Jasco V-530 UV spectrophotometer at pH = 7. High-resolution MS spectra were recorded on a Bruker APEX II FT-ICR mass spectrometer using electrospray ionization (ESI) technique in positive mode. Optical rotations were determined on a Jasco polarimeter using a 1 dm cell at 25 °C; concentrations are in g/100 mL. IR spectra were recorded on a Jasco FT-IR 430 spectrophotometer. Column chromatography was performed by using silica gel 60 (70–230 mesh ASTM, Merck, Vimodrone (MI), Italy). Analytical TLC analyses were performed using F254 silica gel plates (0.2 mm thick, Merck). TLC spots were detected under UV light (254 nm).

*9-(2',3'-O-Isopropylidene-4',5'-di-O-acetyl-d-ribityl)hypoxanthine* (**2**). Compound **1** (200 mg, 0.64 mmol) [34] was dissolved in a solution of Ac_2_O in pyridine (4.0 mL, 4:6, v/v) and the mixture was stirred at room temperature for 16 h (TLC monitoring: CHCl_3_/MeOH, 9:1). The solvents were removed under reduced pressure to afford compound **2** that was used for the next reaction step without further purification. Amorphous white solid (99%, 252 mg); ^1^H-NMR (CD_3_OD) δ 8.06 (s, 1H, 2-H), 8.04 (s, 1H, 8-H), 5.26–5.18 (m, 1H, 4'-H), 4.66 (dd, *J* = 12.3, 2.3 Hz, 1H, 5'-H_a_), 4.63–4.57 (m, 1H, 2'-H), 4.51–4.43 (complex signal, 2H, 3'-H and 1'-H_a_), 4.22 (dd, *J* = 13.9, 10.4 Hz, 1H, 1'-H_b_), 4.14 (dd, *J* = 12.3, 5.2 Hz, 1H, 5'-H_b_), 2.12 (s, 3H, CH_3_CO), 2.07 (s, 3H, CH_3_CO), 1.54 (s, 3H, isopropylidene), 1.30 (s, 3H, isopropylidene); ^13^C-NMR (CDCl_3_) δ 170.7, 170.0, 158.5, 149.0, 145.1, 141.5, 123.4, 110.1, 75.4, 74.1, 68.9, 63.0, 44.0, 27.9, 25.4, 21.1, 20.8; *m*/*z* 417.1390 (HRESIMS) ([M+Na]^+^, C_17_H_22_N_4_NaO_7_ requires 417.1386). IR (neat) ν_max_ 2,854, 1,740, 1,686, 1,219, 1,069 cm^−1^. UV (MeOH) λ_max_ 268 nm.

*1-(2,4-Dinitrophenyl)-9-(2',3'-O-Isopropylidene-4',5'-di-O-acetyl-d-ribityl)hypoxanthine* (**3**). A mixture of **2** (150 mg, 0.38 mmol), DNClB (453 mg, 1.5 mmol), and K_2_CO_3_ (207 mg, 1.5 mmol) was suspended in anhydrous DMF (5.0 mL) and stirred at 80 °C for 3 h. The reaction was monitored by TLC (CHCl_3_/MeOH, 95:5). After cooling, the mixture was filtered and the solid was washed with CHCl_3_. The filtrates and washings, collected and evaporated to dryness, were applied on a silica gel column eluted with increasing amounts of MeOH in CHCl_3_(from 0 to 5%) to give pure **3**, consisting of a 1:1 mixture of atropisomers at the N(1)-phenyl bond. Pale yellow amorphous solid (95%, 202 mg); ^1^H-NMR (CDCl_3_) δ 9.02 (d, *J* = 2.3 Hz, 1H, H-3 DNP), 9.00 (d, *J* = 2.3 Hz, 1H, H-3 DNP), 8.68 (bd, *J* = 8.5 Hz, 2H, 2 × H-5 DNP), 8.29 (bs, 1H, 2-H), 8.23 (bs, 1H, 2-H), 8.10 (bs, 1H, 8-H), 8.08 (bs, 1H, 8-H), 7.91–7.80 (m, 2H, 2 × H-6 DNP), 5.24–5.16 (m, 2H, 4'-H), 4.69 (dd, *J* = 12.4, 2.2 Hz, 1H, 5'-H_a_), 4.64–4.52 (complex signal, 4H, 2 × 1'-H_a_, 2 × 2'-H), 4.50–4.42 (m, 2H, 2 × 3'-H), 4.22–4.10 (complex signal, 4H, 2 × 1'-H_b_, 2 × 5'-H_b_), 2.18 (s, 3H, CH_3_CO), 2.15 (s, 3H, CH_3_CO), 2.11 (s, 6H, 2 × CH_3_CO), 1.58 (s, 3H, CH_3_), 1.56 (s, 3H, CH_3_); 1.37 (s, 3H, CH_3_), 1.34 (s, 3H, CH_3_); ^13^C-NMR (CDCl_3_) δ 170.7, 169.9, 154.2, 148.1, 147.2, 146.1, 145.7, 141.2, 135.3, 132.1, 129.0, 124.5, 121.2, 110.4, 75.2, 74.1, 68.8, 62.7, 44.5, 27.8, 25.3, 21.0, 20.7; *m*/*z* 583.1406 (HRESIMS) ([M+Na]^+^, C_23_H_24_N_6_NaO_11_, requires 583.1401). IR (neat) ν_max_ 3,445, 3,346, 3,099, 1,739, 1,546, 1,341, 1,220, 1,072 cm^−1^. UV (MeOH) λ_max_ 244 nm.

*5-Amino-1-(2',3'-O-Isopropylidene-d-ribityl)-1-H-imidazole-4-carboxamide* (**4**). Compound **3** (100 mg, 0.18 mmol) was dissolved in DMF (2.0 mL) and then EDA (0.24 mL, 3.6 mmol) was added. The mixture was stirred at 50 °C for 16 h (TLC monitoring: CHCl_3_/MeOH, 8:2) and then the solvents were removed under reduced pressure. The crude was applied on a silica gel column eluted with increasing amounts of MeOH in CHCl_3_ (from 0 to 10%) to afford pure **4**. Pale yellow amorphous solid (71%, 38 mg); ^1^H-NMR (CD_3_OD) δ 7.20 (s, 1H, 2-H), 4.46 (m, 1H, 2'-H), 4.42–4.34 (m, 1H, 1'-H_a_), 4.22–4.13 (m, 1H, 3'-H), 3.98 (dd, *J* = 13.6, 10.5 Hz, 1H, 1'-H_b_), 3.82–3.70 (complex signal, 2H, 4'-H and 5'-H_a_), 3.61 (dd, *J* = 11.2, 5.1 Hz, 5'-H_b_), 1.49 (s, 3H, isopropylidene), 1.30 (s, 3H, isopropylidene); ^13^C-NMR (CD_3_OD) δ 167.2, 145.7, 133.1, 113.1, 110.4, 77.7, 77.4, 70.9, 65.1, 45.5, 28.2, 25.5; *m*/*z* 323.1328 (HRESIMS) ([M+Na]^+^, C_12_H_20_N_4_NaO_5_, requires 323.1331). IR (neat) ν_max_ 3,428, 2,961, 1,665, 1,591, 1,262, 1,031, 801 cm^−1^. UV (MeOH) λ_max_ 265 nm.

*5-Amino-1-(d-ribityl)-1-H-imidazole-4-carboxamide* (**5**). Compound **4** (20 mg, 0.066 mmol) was dissolved in 1.0 mL of a solution of TFA in H_2_O (1:9, v/v) at 0 °C. After 15 min the cold bath was removed and the mixture was shaken at room temperature for additional 2 h (TLC monitoring: CHCl_3_/MeOH, 7:3). The solvents were evaporated under reduced pressure and the crude was purified by HPLC (see General methods, t_R_ = 17.7 min) to afford pure **5**. Oil (99%, 17 mg). [α]_D_ −9.7 (*c =* 0.2, CH_3_OH). ^1^H-NMR (CD_3_OD) δ 7.21 (s, 1H, 2-H), 4.14–3.95 (complex signal, 3H, 1-H_a,b_ and 2'-H), 3.80–3.70 (complex signal, 2H, 5'-H_a_ and 3'-H), 3.67–3.60 (dd, *J* = 10.7, 5.3 Hz, 1H, 5'-H_b_), 3.50–3.45 (m, 1H, 4'-H); ^13^C-NMR (CD_3_OD) δ 167.3, 146.2, 133.4, 112.9, 74.4, 73.6, 72.8, 64.4, 47.2; *m*/*z* 283.1021 (HRESIMS) ([M+Na]^+^, C_9_H_16_N_4_NaO_5_, requires 283.1018). IR (neat) ν_max_ 3,341, 2,917, 1,635, 1,588, 1,265, 1,029, 796 cm^−1^. UV (MeOH) λ_max_ 265 nm.

*1-(5-Hydroxypentyl)-9-(2',3'-O-Isopropylidene-d-ribityl)hypoxanthine* (**6**). Compound **3** (100 mg, 0.18 mmol) was dissolved in DMF (2.0 mL) and then 5-aminopentan-1-ol (186 mg, 1.8 mmol) was added. The mixture was stirred at 50 °C for 16 h (TLC monitoring: CHCl_3_/MeOH, 8:2) and then the solvent was removed under reduced pressure. The crude was applied on a silica gel column eluted with increasing amounts of MeOH in CHCl_3_ (from 0 to 10%) to afford pure **6**. Oil (85%, 61 mg). ^1^H-NMR (CD_3_OD) δ 8.27 (s, 1H, 2-H), 8.07 (s, 1H, 8-H), 4.72 (dd, *J* = 14.0, 2.4 Hz, 1H, 1'-H_a_), 4.61–4.54 (m, 1H, 2'-H), 4.29 (dd, *J* = 14.0, 10.7 Hz, 1H, 1'-H_b_), 4.26–4.19 (m, 1H, 3'-H), 4.08 (t, *J* = 7.3 Hz, 2H, CH_2_N), 3.85–3.77 (complex signal, 2H, 4'-H, 5'-H_a_), 3.64 (dd, *J* = 11.5, 5.6 Hz, 1H, 5'-H_b_), 3.54 (t, *J* = 6.4 Hz, 2H, CH_2_O), 1.83–1.71 (m, 2H, CH_2_), 1.62–1.51 (m, 2H, CH_2_), 1.48 (s, 3H, CH_3_), 1.46–1.36 (m, 2H, CH_2_), 1.26 (s, 3H, CH_3_); ^13^C-NMR (CD_3_OD) δ 158.2, 149.4, 149.2, 143.1, 124.2, 110.6, 77.3, 77.0, 70.9, 65.3, 62.5, 47.8, 45.8, 33.1, 30.5, 28.4, 25.7, 23.9; *m*/*z* 419.1911 (HRESIMS) ([M+Na]^+^, C_18_H_28_N_4_NaO_6_, requires 419.1907). IR (neat) ν_max_ 3,345, 2,939, 1,682, 1,547, 1,366, 1,215, 1,070 cm^−1^. UV (MeOH) λ_max_ 250 nm.

*5-Amino-1-(2',3'-O-Isopropylidene-d-ribityl)-N-(5-hydroxypentyl)-1-H-imidazole-4-carboxamide* (**7**). Compound **6** (50 mg, 0.13 mmol) was dissolved in EtOH (2.0 mL) and then a 5 M solution of NaOH (1.4 mL) was added. The mixture was refluxed and the reaction was monitored by UV spectrophotometry. After 5 h the disappearance of the purine band at *λ*_max_ 249 nm and the concomitant appearance of the imidazole band at *λ*_max_ 268 nm confirmed the end of the reaction. The reaction was quenched with 1.4 mL of a 5 M solution of NH_4_Cl and the solvents were removed under reduced pressure. The crude was adsorbed on silica gel and applied on a silica gel column eluted with increasing amounts of MeOH in CHCl_3_ (from 0 to 10%) affording pure **7**. Oil (80%, 40 mg). ^1^H-NMR (CD_3_OD) δ 7.21 (s, 1H, 2-H), 4.50–4.42 (m, 1H, 2'-H), 4.42–4.34 (dd, *J* = 14.7, 1.9 Hz, 1H, 1'-H_a_), 4.20–4.14 (m, 1H, 3'-H), 3.98 (dd, *J* = 14.7, 10.3 Hz, 1H, 1'-H_b_), 3.79 (dd, *J* = 11.3, 2.6 Hz, 1H, 5'-H_a_), 3.76–3.70 (m, 1H, 4'-H), 3.65–3.59 (dd, *J* = 11.3, 5.3 Hz, 1H, 5'-H_b_), 3.56 (t, *J* = 6.5 Hz, 2H, CH_2_O), 3.32 (t, *J* = 7.0 Hz, 2H, CH_2_N, partially covered by solvent signal), 1.66–1.52 (m, 4H, 2 × CH_2_), 1.49 (s, 3H, CH_2_), 1.48–1.39 (m, 2H, CH_2_), 1.30 (s, 3H, CH_3_); ^13^C-NMR (CD_3_OD) δ 166.9, 144.8, 133.0, 113.8, 110.4, 77.7, 77.3, 71.0, 65.2, 62.8, 45.5, 39.6, 33.4, 30.7, 28.2, 25.5, 24.3; *m*/*z* 409.2066 (HRESIMS) ([M+Na]^+^, C_17_H_30_N_4_NaO_6_, requires 409.2063). IR (neat) ν_max_ 3,324, 2,928, 1,621, 1,554, 1,245, 1,218, 1,067 cm^−1^. UV (MeOH) λ_max_ 265 nm.

*5-Amino-1-(d-ribityl)-N-(5-hydroxypentyl)-1-H-imidazole-4-carboxamide* (**8**). Compound **7** (20 mg, 0.052 mmol) was dissolved in 1.0 mL of a solution of TFA in H_2_O (1:9, v/v) at 0 °C. After 15 min the cold bath was removed and the mixture was shaken at room temperature for additional 2 h (TLC monitoring: CHCl_3_/MeOH, 7:3). The solvents were removed under reduced pressure and the crude was adsorbed on silica gel and applied on a silica gel column eluted with increasing amounts of MeOH in CHCl_3_ (from 0 to 20%) affording pure **8**. Oil (99%, 18 mg). [α]_D_ −33.6 (*c =* 0.1, CH_3_OH). ^1^H-NMR (CD_3_OD) δ 7.19 (s, 1H, 2-H), 4.14–3.92 (complex signal, 3H, 1'-H_a,b_ and 2'-H), 3.79–3.69 (complex signal, 2H, 5'-H_a_ and 3'-H), 3.67–3.59 (dd, *J* = 10.7, 5.4 Hz, 5'-H_b_), 3.59–3.52 (t, *J* = 6.5 Hz, 2H, CH_2_OH), 3.50–3.43 (m, 1H, 4'-H), 3.32 (t, *J* = 7.0 Hz, 2H, CH_2_N, partially covered by solvent signal), 1.66–1.52 (m, 4H, 2 × CH_2_), 1.49–1.38 (m, 2H, CH_2_); ^13^C-NMR (CD_3_OD) δ 166.8, 145.3, 133.3, 113.6, 74.4, 73.6, 72.9, 64.4, 62.8, 47.1, 39.6, 33.3, 30.7, 24.2; *m*/*z* 369.1753 (HRESIMS) ([M+Na]^+^, C_14_H_26_N_4_NaO_6_, requires 369.1750). IR (neat) ν_max_ 3,341, 2,923, 1,621, 1,566, 1,253, 1,051 cm^−1^. UV (MeOH) λ_max_ 265 nm.

*5-Amino-1-(4-hydroxybutyl)-1-H-imidazole-4-carboxamide* (**10**). Compound **9** [39] (50 mg, 0.12 mmol) was dissolved in DMF (1.0 mL) and then EDA (0.16 mL, 2.4 mmol) was added. The mixture was stirred at 50 °C for 16 h (TLC monitoring: CHCl_3_/MeOH, 7:3) and then the solvents were removed under reduced pressure. The crude was applied on a silica gel column eluted with increasing amounts of MeOH in CHCl_3_ (from 0 to 20%) to afford pure **10**. Amorphous solid (69%, 16 mg). ^1^H-NMR (CD_3_OD) δ 7.19 (s, 1H, 2-H), 3.89 (t, *J* = 7.1 Hz, 2H, CH_2_N), 3.58 (t, *J* = 6.2 Hz, 2H, CH_2_O), 1.87–1.74 (m, 2H, CH_2_), 1.60–1.47 (m, 2H, CH_2_); ^13^C-NMR (CD_3_OD) δ 167.3, 145.5, 132.1, 112.9, 62.3, 44.3, 30.3, 27.1; *m*/*z* 221.1017 (HRESIMS) ([M+Na]^+^, C_8_H_14_N_4_NaO_2_, requires 221.1014). IR (neat) ν_max_ 3,308, 1,632, 1,555, 1,262, 1,081 cm^−1^. UV (MeOH) λ_max_ 268 nm.

*9-(4-Acetoxybutyl)-1-(5-hydroxypentyl)hypoxanthine* (**11**). Compound **9** (50 mg, 0.12mmol) was dissolved in DMF (1.0 mL) and then 5-aminopentan-1-ol (124 mg, 1.2 mmol) was added. The mixture was stirred at 50 °C for 16 h (TLC monitoring: CH_2_Cl_2_/MeOH, 9:1) and then the solvent was removed under reduced pressure. The crude was applied on a silica gel column eluted with increasing amounts of MeOH in CH_2_Cl_2_ (from 0 to 10%) to afford pure **11**. Oil (78%, 31 mg). ^1^H-NMR (CD_3_OD) δ 8.31 (s, 1H, 2-H), 8.07 (s, 1H, 8-H), 4.27 (t, *J* = 7.1 Hz, 2H, CH_2_OAc), 4.14–4.06 (complex signal, 4H, 2 × CH_2_N), 3.56 (t, *J* = 6.4 Hz, 2H, CH_2_O), 2.01 (s, 3H, CH_3_), 1.99–1.91 (m, 2H, CH_2_), 1.86–1.75 (m, 2H, CH_2_), 1.71–1.54 (complex signal, 4H, 2 × CH_2_); ^13^C-NMR (CD_3_OD) δ 172.2, 158.3, 149.4 (2C), 142.4, 124.6, 64.8, 62.6, 47.8, 44.6, 33.1, 30.5, 27.8, 26.7, 23.9, 20.7; *m*/*z* 359.1692 (HRESIMS) ([M+Na]^+^, C_16_H_24_N_4_NaO_4_, requires 359.1695). IR (neat) ν_max_ 3,434, 2,923, 1,679, 1,575, 1,259, 1,031, 796 cm^−1^. UV (MeOH) λ_max_ 249 nm.

*5-Amino-1-(4-hydroxybutyl)-N-(5-hydroxypentyl)-1-H-imidazole-4-carboxamide* (**12**). Compound **11** (20 mg, 0.059 mmol) was dissolved in EtOH (1.0 mL) and then 0.6 mL of a 5 M solution of NaOH were added. The mixture was refluxed and the reaction was monitored by UV spectrophotometry. After 5 h the disappearance of the purine band at *λ*_max_ 249 nm and the concomitant appearance of the imidazole band at *λ*_max_ 268 nm confirmed the end of the reaction. The reaction was quenched with 0.6 mL of a 5 M solution of NH_4_Cl and the solvents were removed under reduced pressure. The crude was purified by HPLC (t_R_ = 25.8 min, see General Methods) affording pure **12**. Oil (75%, 13 mg). ^1^H-NMR (CD_3_OD) δ 7.20 (s, 1H, 2-H), 3.9 (t, *J* = 7.2 Hz, 2H, CH_2_NC=C), 3.6 (t, *J* = 6.4 Hz, 2H, CH_2_O), 3.50 (t, *J* = 6.5 Hz, 2H, CH_2_O), 3.31 (CH_2_NC=O, covered by solvent signal), 1.87–1.76 (m, 2H, CH_2_), 1.65–1.49 (m, 6H, 3 × CH_2_), 1.48–1.39 (m, 2H, CH_2_); ^13^C-NMR (CD_3_OD) δ 166.9, 144.6, 132.0, 113.4, 62.8, 62.3, 44.2, 39.5, 33.3, 30.7, 30.3, 27.1, 24.3; *m*/*z* 307.1749 (HRESIMS) ([M+Na]^+^, C_13_H_24_N_4_NaO_3_, requires 307.1746). IR (neat) ν_max_ 3,324, 1,621, 1,564 cm^−1^. UV (MeOH) λ_max_ 268 nm.

## 4. Conclusions

We have here reported useful synthetic procedures to introduce alternative moieties (d-ribityl or 4-hydroxybutyl) into the ribose portion of the AICA-riboside. The obtained AICAR analogues have the imidazole (AICA) without modifications (compounds **5** and **10**) or bear the 5-hydroxypentyl chain on a carboxamide function (compounds **8** and **12**). We believe that these synthetic pathways could enlarge the toolbox of the reactions operating on the AICA riboside (acadesine) and could furnish new “tuneable” AICAR analogues in terms of their molecular size, flexibility and hydrogen bond formation for their interactions with metabolic enzymes. Works are in progress to evaluate their activity into AMPK-dependent and –independent processes in order to better understand the metabolic pathways involving AICAR and ZMP.
